# RSM- and ANN-Based Optimization of Bioactive Anthocyanin and Phenolic Compound Recovery from *Rosa damascena* Flowers Using Natural Deep Eutectic

**DOI:** 10.3390/antiox15060656

**Published:** 2026-05-22

**Authors:** Georgia D. Ioannou, Atalanti Christou, Michaella Pieri, Panayiota Piri, George Botsaris, Vlasios Goulas

**Affiliations:** Department of Agricultural Sciences, Biotechnology and Food Science, Cyprus University of Technology, 3603 Lemesos, Cyprus; georgia.ioannou@cut.ac.cy (G.D.I.); mv.pieri@edu.cut.ac.cy (M.P.); ps.piri@edu.cut.ac.cy (P.P.); george.botsaris@cut.ac.cy (G.B.)

**Keywords:** *Rosa damascena*, Damask rose, natural deep eutectic solvents, ultrasound-assisted extraction, response surface methodology, artificial neural networks, anthocyanins, antioxidants

## Abstract

The present study investigated the use of natural deep eutectic solvents (NaDESs) combined with ultrasound-assisted extraction (UAE) for the efficient recovery of anthocyanins and antioxidant phenolics from Damask rose (DR). A wide range of environmentally friendly solvents was screened, and choline chloride–propylene glycol (ChCl-PG) was identified as the most effective extraction medium. The extraction conditions were optimized using response surface methodology (RSM) and artificial neural network (ANN) models to maximize anthocyanin and phenolic contents, as well as antioxidant activity. Under the optimal parameters, the DR extracts exhibited relatively high levels of bioactive compounds, including total anthocyanin content of 5.2–5.3 mg cyanidin-3-glucoside equivalents g^−1^ sample, total phenolic content of 63.4–64.2 mg gallic acid equivalents g^−1^ sample, along with substantial antioxidant potential (DPPH: 68.2–68.8% inhibition, FRAP: 581.6–591.9 μmol Trolox equivalents g^−1^ sample). Chromatographic analysis of the optimum extract revealed cyanidin as the predominant anthocyanidin in DR, and its stability was further evaluated, revealing improved preservation under dark conditions at lower temperatures over a 15-day storage period. Moreover, the IC_50_ values confirmed antimicrobial effects against the tested foodborne pathogens. Furthermore, the inhibitory effect of the DR extract remained stable against *S. aureus* and *S. cerevisiae* throughout the storage period. Overall, the findings demonstrate that NaDES-UAE is a promising and sustainable approach for obtaining anthocyanin-rich DR extracts with antioxidant and antimicrobial potential.

## 1. Introduction

*Rosa damascena* Mill, frequently referred to as the Damask rose (DR), represents an industrially significant species within the Rosaceae family. *R. damascena* is a small, deciduous shrub, characterized by its pale pink flowers, and it is widely cultivated across numerous countries in Europe and West Asia [1]. This species possesses considerable commercial significance, being extensively utilized in a range of industries, including the production of flavoring agents, perfumery components, cosmetics, pharmaceuticals, and aromatics [2]. Rose flowers are routinely processed for the development of diverse commercial products, including rose essential oil, rose water, dried flowers, rose hips, rose concrete, and rose absolute, with the volatile components typically isolated via techniques such as hydrodistillation and solvent extraction [3]. Furthermore, *R. damascena* is also recognized as a significant source of polyphenolic compounds, including phenolic acids, flavonoids, tannins, and anthocyanins [4].

The anthocyanins, water-soluble flavonoid pigments, based on the flavylium cation structure, are responsible for the coloration of DR petals. The predominant anthocyanins identified in the DR include glucoside forms of cyanidin, pelargonidin, peonidin, and malvidin. The specific chromatic characteristics exhibited by these pigments are fundamentally determined by their respective hydroxylation and methylation patterns on the B-ring of the flavonoid structure. These aglycones are primarily stabilized through glycosylation, most commonly involving glucose moieties attached at the C-3 and/or C-5 positions of the skeleton, forming 3-glucosides and 3,5-diglucosides. Research has also differentiated the pigment profile between rose types, revealing that the cyanidin and peonidin 3-glucosides and 3,5-diglucosides are characteristic of wild species, whereas the pelargonidin 3-glucoside and 3,5-diglucoside forms are notably more prevalent in cultivated varieties, suggesting a chemotypic shift driven by selective breeding for desired color traits. In the broader food science context, anthocyanins are functionally employed as natural colorants and as natural preservatives and flavor scavengers, primarily by mitigating environmental stress factors to protect sensitive food ingredients during storage and transportation [5]. Specific to DR anthocyanins, their established value extends to applications as antimicrobial and antioxidant agents when incorporated into polymeric films for active food packaging. Furthermore, their pH-dependent color changes enable their successful integration as colorimetric pH indicators in smart packaging materials [6,7].

The extraction of anthocyanins from plant matrices has relied on conventional extraction methods, which typically employ organic solvents such as alcohols, acetone, or their mixtures with water, often necessitating the application of heating. However, the field is undergoing a significant transformation, marked by a progressive move toward emerging extraction techniques, such as supercritical fluid extraction, ultrasound-assisted extraction (UAE), and microwave-assisted extraction, which are increasingly favored for their enhanced efficiency and improved greenness [8]. In this context, there is also a growing preference for alternative, “green” solvents. Among various solvent systems, natural deep eutectic solvents (NADESs) represent sustainable alternatives to hazardous organic solvents due to their favorable safety profiles and environmental compatibility. Moreover, NADESs demonstrate the capability to improve extraction yields and selectivity for hydrophilic and thermolabile phytochemicals like anthocyanins, often without requiring harsh conditions such as high temperatures or extreme pH changes. Their derivation from natural components further positions NADESs as highly suitable for applications in the food and pharmaceutical industries [9,10]. They are a novel class of green solvents derived entirely from natural components, primarily plant-based primary metabolites such as amino acids, sugars, sugar alcohols, and organic acids. They are synthesized simply by mixing a hydrogen bond donor (HBD) and a hydrogen bond acceptor (HBA) in specific molar ratios. These mixtures form a eutectic mixture characterized by a significantly lower melting point than its individual starting materials. The specific nature and proportions of these initial components allow NADESs to exhibit a diverse range of physicochemical properties, which dictate their specific applications [11]. Despite their advantages, their relatively high viscosity often poses a barrier to efficient mass transfer, and current limitations in large-scale applicability remain a significant hurdle for industrial-scale integration.

Current methodologies for the extraction of anthocyanins from DR predominantly utilize maceration and UAE, employing conventional solvents such as acidified water, acidified alcohols, and aqueous alcoholic mixtures [12,13,14]. The present study introduces several novel elements to the field of DR valorization. Notably, this work transcends typical optimization by providing a rigorous comparative evaluation of RSM and ANN modeling, an approach that remains unexplored for DR, to navigate the nonlinear complexities of NaDES-based systems. Unlike existing literature that often focuses on essential oils or limited solvent systems, this study represents a systematic effort to optimize the recovery of the entire bioactive phenolic and anthocyanin fraction using an extensive library of 38 NaDESs. By integrating long-term anthocyanin and antimicrobial stability studies against foodborne pathogens into the optimization framework, this research offers a holistic approach that bridges the gap between green extraction technology and the practical requirements of the food and pharmaceutical industries.

## 2. Materials and Methods

### 2.1. Plant Material and Chemicals

The DR flowers were kindly supplied by The Rose Factory by Tsolakis (Agros, Cyprus) at the end of April 2025. The flowers were lyophilized and ground to a fine powder using an electric mill. The dried samples were vacuum-packed and stored at −20 °C until further extraction and analysis.

All chemicals were of analytical reagent grade. The analytical standards of delphinidin chloride, cyanidin chloride, petunidin chloride, pelargonidin chloride, peonidin chloride, and malvidin chloride were purchased from Cayman Chemical (Ann Arbor, MI, USA), while gallic acid, catechin hydrate, tannic acid, and trolox standards were obtained from Sigma-Aldrich (Steinheim, Germany). HPLC-grade acetonitrile (ACN), methanol (MeOH), and ethanol (EtOH) were provided by Supelco (Bellefonte, PA, USA). Trifluoroacetic acid (TFA), used to acidify the HPLC mobile phase, was purchased by Merck (Darmstadt, Germany). Folin–Ciocalteu reagent, sodium carbonate (Na_2_CO_3_), hydrochloric acid (HCl), potassium chloride (KCl), 2,2-diphenyl-1-picrylhydrazyl (DPPH), acetic acid, 2,4,6-tris(2-pyridyl)-s-triazine (TPTZ), potassium iodate (KIO_3_), vanillin, sorbitol (Sor), xylitol (Xyl), D-(−)-fructose (Fru), sucrose (Suc), L-(+)-lactic acid (LA), malic acid (MA), tartaric acid (TA) and oxalic acid (OA) were also acquired from Sigma-Aldrich (Steinheim, Germany). Sodium nitrite (NaNO_2_), sodium acetate trihydrate (C_2_H_3_NaO_2_^.^3H_2_O), aluminum chloride (AlCl_3_), propylene glycol (PG), glycerol (Gly), D-(+)-glucose (Glu), and citric acid (CA) were obtained from Sharlau Chemie (Barchelona, Spain). Choline chloride (ChCl), betaine (Bet), proline (Pro), glycine (Glyc), and alanine (Ala) were acquired from BLD Pharmatech (Reinbek, Germany). Sodium hydroxide (NaOH) was obtained from Merck (Darmstadt, Germany), while iron (III) chloride hexahydrate (FeCl_3_^.^6H_2_O) was purchased from Honeywell (Charlotte, NC, USA).

### 2.2. Preparation and Characterization of NaDESs

Thirty-eight distinct NaDESs were successfully prepared utilizing ChCl, Bet, Pro, Glyc, and Ala as HBAs in combination with various HBDs, including polyols (PG, Gly, Sor, Xyl), organic acids (MA, LA, CA, TA, OA), and sugars (Glu, Fru, Suc) (Table 1). The preparation involved mixing the HBA and HBD components at specific molar ratios in sealed flasks, followed by stirring and heating at 80 °C for a duration ranging from 30 to 120 min, until a transparent, colorless liquid was formed. Upon cooling, the resulting eutectic mixtures were subsequently diluted with 20% water to achieve a reduction in viscosity and a concurrent enhancement of their solvation power [11]. For stability, the prepared NaDESs were then stored in sealed vials within a desiccator, maintained in the dark at room temperature.

Initially, the pH of the NaDESs (containing 20% *w*/*w* water) was measured using an Edge^®^blu digital pH meter (Edge^®^blu, Hanna Instruments, Woonsocket, RI, USA). Dynamic viscosity was determined across a range of rotation rates (5–100 rpm) using a Brookfield LVDV-E viscometer (Brookfield Engineering Laboratories, Middleboro, MA, USA) equipped with an S03 spindle. Both pH and viscosity characterizations were conducted at a controlled temperature of 25 °C. To investigate intermolecular interactions and hydrogen bond formation, ATR-FTIR spectra of the NaDESs and their precursors were recorded on an Agilent Cary 630 ATR-FTIR spectrometer (Agilent Technologies, Santa Clara, CA, USA). Spectra were collected over the 4000–600 cm^−1^ range with a resolution of 32 cm^−1^ and an accumulation of 4 scans.

**Table 1:** Viscosity and pH values of the prepared NaDESs.

HBA	HBD	NaDES Abbreviation	Molar Ratio	Water Content 20% *w*/*w*	
				Viscosity (cP)	pH
Choline chloride	Propylene glycol	ChCl-PG	1:2	45	4.95
	Glycerol	ChCl-Gly	1:2	58	4.32
	Sorbitol	ChCl-Sor	1:1	160	5.46
	Xylitol	ChCl-Xyl	1:1	95	6.63
	Glucose	ChCl-Glu	2:1	153	5.06
	Frustose	ChCl-Fru	2:1	134	4.58
	Sucrose	ChCl-Suc	5:2	297	5.43
	Malic acid	ChCl-MA	1:1	108	<1
	Lactic acid	ChCl-LA	1:1	48	<1
	Citiric acid	ChCl-CA	1:1	128	<1
	Tartaric acid	ChCl-TA	1:1	273	<1
Betaine	Propylene glycol	Bet-PG	1:2	66	7.92
	Glycerol	Bet-Gly	1:2	105	7.15
	Sorbitol	Bet-Sor	1:1	592	7.45
	Xylitol	Bet-Xyl	1:1	231	7.55
	Glucose	Bet-Glu	2:1	1252	7.69
	Frustose	Bet-Fru	2:1	730	7.64
	Sucrose	Bet-Suc	5:2	3057	7.76
	Malic acid	Bet-MA	1:1	605	2.97
	Lactic acid	Bet-LA	1:1	101	3.9
	Citiric acid	Bet-CA	1:1	542	2.33
	Tartaric acid	Bet-TA	1:1	1948	2.17
Proline	Glycerol	Pro-Gly	1:2	132	6.73
	Sorbitol	Pro-Sor	1:1	1102	7.01
	Xylitol	Pro-Xyl	1:1	666	6.96
	Glucose	Pro-Glu	1:1	2817	5.89
	Frustose	Pro-Fru	1:1	2161	6.85
	Sucrose	Pro-Suc	2:1	7058	7.1
	Malic acid	Pro-MA	1:1	938	2.33
	Lactic acid	Pro-LA	1:2	73	2.6
	Citiric acid	Pro-CA	1:1	922	1.93
	Oxalic acid	Pro-OA	1:1	80	<1
Glycine	Malic acid	Glyc-MA	1:1	1257	2.35
	Lactic acid	Glyc-LA	1:3	66	2.28
	Citiric acid	Glyc-CA	1:2	4810	1.57
	Tartaric acid	Glyc-TA	1:1	4930	1.75
Alanine	Citiric acid	Ala-CA	1:1	1569	1.97
	Tartaric acid	Ala-TA	1:1	11,850	1.87

HBA: Hydrogen bond acceptor, HBD: Hydrogen bond donor.

### 2.3. Optimization of UAE Using RSM and ANN

#### 2.3.1. Process Variables for UAE

The UAE procedure was performed using an ultrasonic bath (UCl-50, 35 KHz, Raypa-R. Espinar, S.L., Terrassa, Spain). Aliquots of DR (0.2 g) were dissolved with NaDESs at different volumes (2–6 mL) and water concentrations (10–50% *w*/*w*). Each sample was thoroughly homogenized using a vortex prior to ultrasonic extraction. Based on the developed experimental design or screening evaluation, the solutions were subjected to ultrasound irradiation at different temperatures (10, 20, 30, 40, and 50 °C) and for various periods (10, 20, 30, 40, and 50 min). Following the ultrasonic treatment, the resulting mixtures were centrifuged at 2500 rpm for 20 min. The supernatants were collected and stored at 4 °C until further analysis. All extracts were prepared in triplicate.

#### 2.3.2. Statistical Optimization via RSM

A central composite design (CCD) under RSM was applied to determine optimal UAE conditions for phenolic antioxidants and anthocyanin extraction. Four independent variables were evaluated for their impact on extraction efficiency: extraction time (A, 10–50 min), extraction temperature (B, 10–50 °C), solvent-to-solid ratio (C, 10–30 mL g^−1^), and water content in NaDES (D, 10–50% *w*/*w*), with their ranges selected based on preliminary studies, taking into account extraction efficiency and anthocyanin stability. Total anthocyanin content (TAC), antioxidant activity in terms of DPPH inhibition and ferric reducing antioxidant power (FRAP), and total phenolic content (TPC) were selected as the responses of the optimization. The complete CCD, which consisted of 30 combinations including six replicates at the central point, is presented in Table 2. RStudio statistical software was employed for experimental design, model building, and data interpretation.

#### 2.3.3. Artificial Neural Network Modeling

In the present study, a multilayer feed-forward neural network, trained with an error backpropagation algorithm, was also employed to model the UAE of polyphenolic antioxidants. The experimental dataset obtained from the CCD was used to construct the ANN model. In particular, the experimental matrix (consisting of 90 points), previously normalized between 0 and 1, was divided into a training/validation set (80%) and a test set (20%). K-fold cross-validation was employed to train and validate the ANN model, in which the training/validation data were randomly divided into five subsets (k = 5). In each iteration, one subset was used as the validation set, while the remaining subsets served as the training set for model development.

The optimal ANN topology consisted of an input layer with four neurons corresponding to the independent variables (extraction time, temperature, solvent-to-solid ratio, and NaDES water content), a hidden layer with nine neurons, and an output layer comprising four neurons representing the response variables (TAC, TPC, DPPH inhibition, and FRAP). The optimal architecture was identified by evaluating feed-forward networks of varying topologies. The selection criteria focused on minimizing the mean squared error (MSE) and maximizing the coefficient of determination (R^2). A sigmoidal transfer function was implemented in the hidden layer, whereas the output layer utilized a linear activation function.

The established ANN model was finally combined with a genetic algorithm (GA) to optimize the input parameters and to obtain the best output performance. Both ANN modelling and optimization were performed using RStudio statistical software.

### 2.4. Phytochemical Analysis of DR Extracts

TPC of the DR extracts was quantified via a 96-well microplate Folin–Ciocalteu assay. Briefly, 20 µL of the diluted extract was combined with 100 µL of Folin–Ciocalteu reagent (1:4, *v*/*v*) and agitated for 1 min. Following a 4 min incubation, 75 µL of saturated sodium carbonate and 100 µL of 4% (*w*/*v*) sodium hydroxide were added sequentially. The mixture was shaken for 1 min and incubated in the dark at room temperature for 2 h. Absorbance was recorded at 750 nm using a Thermo Scientific Multiskan GO reader (Vaanda, Finland). TPC was calculated using a gallic acid standard curve and expressed as mg gallic acid equivalents (GAE) per g of dry sample [11].

TFC of the DR extracts was determined using the aluminum chloride colorimetric assay. In a 96-well microplate, a 25 μL aliquot of the extract was mixed with 100 μL of distilled water and 10 μL of 50 g L^−1^ sodium nitrite. After 5 min, 15 μL of aluminum chloride solution (100 g L^−1^) was added. Following a subsequent 6 min incubation, the reaction was neutralized by the addition of 50 μL of 1 M sodium hydroxide and 50 μL of distilled water. The plate was then agitated for 30 s, and the absorbance was recorded at 510 nm. TFC was calculated against a catechin standard curve and expressed as mg catechin equivalents (CE) per g of dry sample [11].

TAC was quantified using the pH-differential spectrophotometric method. Extract samples (0.4 mL) were mixed with 3.6 mL of either potassium chloride buffer (0.025 M, pH = 1.0) or sodium acetate buffer (0.4 M, pH = 4.5). Absorbance measurements were performed at 510 and 700 nm. The final content was reported as mg cyanidin-3-glucoside equivalents (C3GE) per g of dry weight using the equation below: cyanidin-3-glucoside (mg/L) = A × MW × DF · 1000/(MA × 1), where A = (A_510_ − A_700_)_pH 1.0_ − (A_510_ − A_700_)_pH 4.5_; MW: molecular weight (449.2); DF: dilution factor; MA: molar absorptivity (26,900) [15].

The vanillin–HCl assay was used to quantify condensed tannins (proanthocyanidins) in the extracts. The assay procedure involved mixing 1 mL of the sample solution with 2.5 mL of a 1% *w*/*v* vanillin solution in methanol, then adding 2.5 mL of 9 mol/L HCl in MeOH. Τhe resulting mixture was then incubated at 30 °C for 20 min, and its absorbance was measured at 500 nm. The results were expressed as mg of catechin hydrate equivalents (CE) per g of dry sample [16].

For the colorimetric determination of total hydrolysable tannin content (THTC), 1 mL of a 10-fold-diluted extract was combined with 5 mL of 2.5% *w*/*v* KIO_3_ in a vial, vortexed briefly for 10 s, and allowed to react for 4 min. The maximum absorbance of the resulting red mixture was measured at 550 nm against a water blank. Calibration was achieved using a range of tannic acid concentrations, and the results were expressed in terms of mg of tannic acid equivalents (TAE) per g of dry sample [17].

### 2.5. Antioxidant Potency of DR Extracts

The radical scavenging activity of the DR was determined by quantifying the bleaching effect on the characteristic purple methanolic solution of DPPH. A 25 μL of the diluted DR extract was mixed with 975 μL of freshly prepared DPPH methanolic solution (60 μM) or extraction solvent (blank). The resulting mixtures were subjected to vigorous agitation and subsequently incubated in darkness at ambient temperature for a period of 30 min. The reduction in the absorbance of the DPPH solution was then measured at a wavelength of 517 nm using a Thermo Scientific Multiskan GO spectrophotometer. The results were presented as the DPPH radical scavenging activity percentage (%) [11].

The antioxidant capacity of the DR extracts was further evaluated by FRAP assay. The working FRAP reagent was freshly prepared by combining 300 mM acetate buffer (pH = 3.6), 10 mM TPTZ solution in 40 mM HCl, and 20 mM FeCl_3_ aqueous solution in a 10:1:1 (*v*/*v*/*v*) ratio, followed by pre-warming to 37 °C. Aliquots of the diluted extracts (150 μL) were reacted with 950 μL of the FRAP reagent and incubated in the dark for 30 min. The absorbance of the resulting colourful complex was recorded at 593 nm using a Multiskan GO spectrophotometer. Results were quantified using a Trolox standard curve and expressed as μmol of Trolox equivalents (TE) per gram of dry sample [11].

### 2.6. pH Sensitivity of DR Extracts

The spectra of properly diluted anthocyanin extracts in pH values ranging from 1.0 to 14.0 were measured over a wavelength range of 400–800 nm after adjusting the pH with 0.1 M HCl or 0.1 M NaOH to the desired values, using a Thermo Scientific Multiskan GO spectrophotometer [13].

### 2.7. Determination of Anthocyanidin Profile of DR by HPLC Analysis

Following UAE optimization, the DR extract was analyzed for anthocyanidin composition. High-performance liquid chromatography was performed on a Waters e2695 platform (Milford, MA, USA) featuring a quaternary solvent delivery system and a thermostated column oven. Detection was carried out via a photodiode array detector. The chromatographic conditions were based on the method of Zhang et al. (2004), incorporating slight modifications [18]. The chromatographic analysis was achieved using a Waters Spherisorb^®^ ODS2 (15 cm, 4.6 mm, 5 μm) column, thermostated at 35 °C at a flow rate of 1 mL/min. The mobile phase consisted of 0.4% TFA in water (mobile phase A) and 0.4% TFA in ACN (mobile phase B). A gradient elution was performed, starting at 85% A for the first 1 min. Mobile phase A then linearly decreased to 78% until 15 min and further to 70% at 30 min. Finally, mobile phase A increased gradually to 85% until 32 min and remained constant until 35 min. The PDA detector was set at 525 nm for the determination of anthocyanidins.

Commercially available anthocyanidin standards were dissolved in acidified methanol containing 2.7 M HCl. After the sonication of each standard, a standard mixture consisting of delphinidin, cyanidin, petunidin, pelargonidin, peonidin, and malvidin was prepared. Calibration curves were constructed by diluting the standard mixture with the acidified methanolic solution to cover the desired concentration range of anthocyanidins.

For the qualitative and quantitative determination of anthocyanidins in DR extracts by HPLC, a hydrolysis step was necessary to convert anthocyanins into their corresponding aglycone forms. The acidic hydrolysis was performed by transferring 0.3 mL HCl 37% (12 M) into 1 mL of the DR extract in a glass bottle and place into a 90 °C water bath for 60 min [19]. Then, samples were cooled, appropriately diluted, and filtered through a 0.45 μm PTFE filter before the HPLC injection.

### 2.8. Determination of Antimicrobial Effects of DR Extract

The antimicrobial activity of DR extract was tested against two Gram-positive bacteria (*Staphylococcus aureus* ATCC 6538, *Listeria monocytogenes* ATCC 4994), two Gram-negative bacteria (*Escherichia coli ATCC 11775, Salmonella enterica* subsp. *Enterica* serovar Enteritidis NCTC 5188), and two yeasts (*Candida albicans* WDCM 00054, *Saccharomyces cerevisiae* ATCC 9763). Each bacterial strain was cultured by inoculating a colony into 10 mL of Mueller–Hinton Broth (MHB; Himedia^®^, Mumbai, India) at 37 °C for 24 h, while *L. monocytogenes* was grown in Brain Heart Infusion broth (BHI, Himedia^®^, Mumbai, India) under the same conditions. Both yeasts were cultured in Dextrose Broth (PDB; Himedia^®^, Mumbai, India) at 25 °C for 24 h. The antimicrobial activity of the extracts was determined via broth microdilution. Each well of a 96-well plate was loaded with 20 µL of extract and inoculated with 100 µL of log-phase culture (10^6^ CFU mL^−1^). Following incubation at 37 °C for 24 h, growth inhibition was screened by monitoring medium turbidity at 600 nm using a microplate reader. The antimicrobial potency of the DR extract was evaluated using the Minimum Inhibitory Concentration (MIC) and the half-maximal inhibitory concentration (IC_50_). The MIC was defined as the lowest extract concentration that prevented visible bacterial growth (turbidity) relative to the negative control. The IC_50_ represented the concentration required to achieve 50% growth inhibition after 24 h. For stability testing, antimicrobial activity was reported as the percentage of bacterial or yeast growth inhibition. All assays were conducted in triplicate to ensure reproducibility.

### 2.9. Stability of DR Extract

To study the effects of temperature, light exposure, and storage time on the anthocyanin content of DR extracts, the extracts obtained under optimal conditions were stored under different conditions, and their anthocyanidin profile was monitored. In particular, the freshly prepared NaDES extract was stored for 15 days under four different conditions: at 4 °C (refrigeration), with and without light exposure (810 lm), and at 25 °C (room temperature), with and without light exposure (810 lm). The stability of anthocyanidins was evaluated by chromatographic analysis of the extract at days 1, 5, 10, and 15 of storage. Results were expressed as % concentration reduction.

### 2.10. Statistical Analysis

All experimental measurements were conducted in triplicate, with results reported as mean value ± standard deviation. Statistical significance was evaluated using one-way analysis of variance (ANOVA), followed by Duncan’s multiple range test to determine differences between means. A confidence level of 95% was applied, and results were considered statistically significant at *p* < 0.05.

In the screening study of NaDESs, the mean values of the obtained data set were subjected to chemometric analysis. The data set consisted of 38 × 5 matrix, in which rows represented the extraction solvents (38 NaDESs) and the columns the TAC, DPPH, FRAP, viscosity, and pH values. To minimize data dimensionality and identify potential clustering of solvents based on extraction efficiency, initial composition, and solvent characteristics, principal component analysis (PCA) was applied as an unsupervised chemometric method. All statistical analyses were conducted using RStudio software (version 2025.05.1-513).

## 3. Results

### 3.1. Green Solvent Screening for Anthocyanin and Antioxidant Recovery

**Figure 1:**
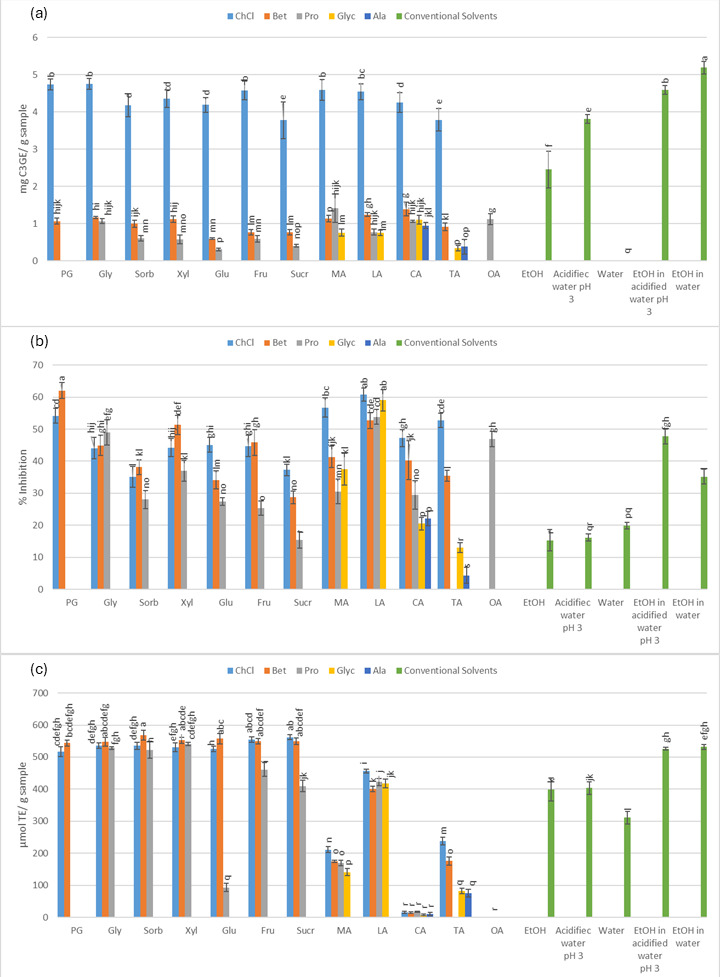
Screening of different green solvents for (**a**) total anthocyanin content (TAC), (**b**) DPPH free radical scavenging, and (**c**) ferric reducing antioxidant power (FRAP). Different lowercase letters for each coloured bar indicate significant differences (*p* < 0.05) according to Duncan’s multiple range test.

Different environmentally friendly extraction media were evaluated for their ability to recover anthocyanins and antioxidants from DR. In total, thirty-eight NaDESs composed of various HBAs and sugars, polyols, and acids as HBDs, along with five conventional green solvents, were screened. The NaDESs examined displayed a broad range of physicochemical properties, particularly viscosity and pH, which affect the extractability of phenolic constituents. All screening experiments were performed at the central point of the independent variables: processing time = 30 min, extraction temperature = 30 °C, solvent-to-solid ratio = 20 mL g^−1^, and percentage of water in NaDES = 30% *w*/*w*. The TAC, along with the antioxidant potential measured by DPPH and FRAP assays, was determined in the resulting DR extracts and used as a key criterion for solvent screening (Figure 1). The extraction yields of TAC, DPPH, and FRAP obtained using different green solvents ranged from 0 to 5.2 mg C3GE g^−1^ sample, 4.4 to 62.1% radical inhibition, and 0 to 568.5 μmol TE g^−1^ sample, respectively.

Overall, the NaDESs outperformed the conventional green solvents significantly. The exception is the TAC values for ethanol in water and ethanol in acidified water, which are comparable to the highest values observed for NaDESs. Polyol-based eutectic mixtures, especially those with propylene glycol and glycerol as HBDs, had the highest extraction efficiencies. These NaDESs consistently yielded extracts with elevated TAC values and notable antioxidant activity. Their relatively high efficiency can be attributed to the favourable polarity and hydrogen-bonding ability of polyols, combined with their relatively low viscosity, which promotes enhanced mass transfer during extraction [20].

Within the NaDES group, ChCl-PG demonstrated the best extraction efficiency. It yielded one of the highest TAC levels among all solvents, while also exhibiting notable radical scavenging and ferric-reducing activities. The superior performance of ChCl-PG is likely due to the balanced hydrogen bonding network formed between choline chloride and propylene glycol, which produces a polar, low-viscosity solvent that efficiently solubilizes anthocyanins and other antioxidant compounds through enhanced solute-solvent hydrogen-bonding interactions [20,21,22]. In addition, specific interactions between the NaDES components and anthocyanins may further contribute to the enhanced extraction efficiency. The chloride ion of choline chloride can interact electrostatically with the positively charged flavylium cation of anthocyanins, while the hydroxyl groups of propylene glycol promote extensive hydrogen-bonding interactions with anthocyanin hydroxyl substituents. Furthermore, the relatively mild acidic environment of NaDES may contribute to the stabilization of the flavylium form of anthocyanins, leading to improved solubility and stability during the extraction procedure [23,24].

Acid-based NaDESs exhibited moderate TAC and DPPH values. Their acidic nature may enhance cell wall disruption and pigment release; however, in the present study, their performance remained below that of the best-performing polyol-based NaDESs [25,26]. On the other hand, sugar-based NaDESs exhibited the weakest overall performance, demonstrating the lowest TAC and DPPH values among all NaDESs. Their high viscosity and reduced mass-transfer capacity, attributed to the strong hydrogen-bonding network between NaDES components, which restricts molecular mobility, may result in limited pigment and antioxidant recovery [27].

**Figure 2:**
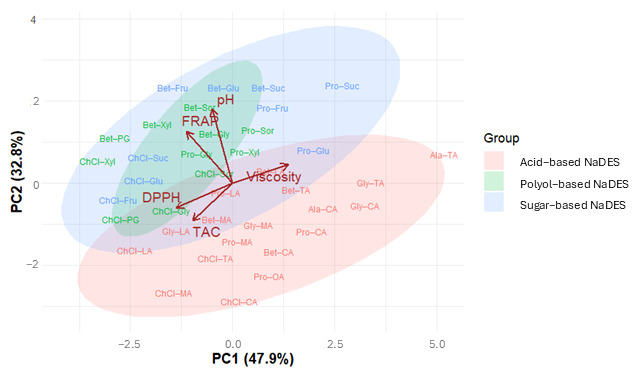
Principal component analysis (PCA) biplot demonstrating the distribution of NaDES extracts according to anthocyanin content, antioxidant activity (DPPH and FRAP), and NaDES physicochemical properties (viscosity and pH).

PCA (Figure 2) provided a comprehensive overview of the behaviour of different NaDES groups, considering both their physicochemical properties (pH and viscosity) and their TAC, DPPH, and FRAP values. The first two principal components (PCs) were able to explain 80.7% of the total variability, with PC_1_ accounting for 47.9% and PC_2_ for 32.8%. The close alignment of TAC, DPPH, and FRAP vectors indicates a strong positive correlation between anthocyanin content and antioxidant activity. In contrast, the viscosity is oriented in the opposite direction, suggesting a negative relationship with extraction efficiency, while pH appears to influence the distribution of acid-based NaDESs along PC_2_. The negative association between viscosity and antioxidant-related variables highlights the importance of solvent fluidity in promoting analyte diffusion and mass transfer from the plant matrix. In addition, pH may influence anthocyanin stability by affecting the equilibrium between their different structural forms, particularly the flavylium cation. Therefore, the combined influence of viscosity and pH appears to play a crucial role in the determination of NaDES extraction efficiency. The polyol-based NaDESs clustered in the region strongly aligned with TAC, DPPH, and FRAP vectors, confirming their superior extraction capacity. The acid-based NaDESs formed a clearly separated group positioned opposite the pH vector, reflecting their characteristically low pH values. At the same time, these NaDESs were positioned closer to the viscosity vector, consistent with their moderate to high viscosity and weaker extraction capacity compared to polyol-based solvents. The sugar-based NaDESs did not form a completely distinct cluster. Several ChCl–sugar solvents appeared near the polyol-based group, resulting in partial overlap between these categories. The abundance of hydroxyl groups in both HBD categories likely enhances hydrogen-bonding interactions with anthocyanins and other antioxidants, improving solubilization. However, these strong interactions may increase solvent viscosity, which can limit mass transfer and partially reduce extraction efficiency compared to lower-viscosity polyol-based NaDESs.

Considering its consistently high extraction capacity and favourable physicochemical properties, ChCl-PG was selected as the optimum green solvent for all subsequent optimization experiments.

### 3.2. Optimization of the Extraction Procedure of DR Using RSM

#### 3.2.1. Model Fitting and Statistical Analysis

Following the identification of the best-performing green solvent for the extraction of anthocyanins and antioxidants from DR, RSM using CCD was applied to evaluate the impact of UAE parameters on extractability and to identify the optimal experimental conditions, including extraction time (A), extraction temperature (B), solvent-to-solid ratio (C), and water content in NaDES (D). The optimization goal was to maximize anthocyanin and antioxidant extractability, with TAC, TPC, DPPH, and FRAP selected as the response variables. The experimental outcomes obtained under the different sets of UAE parameters are presented in Table 2. The results were in the range of 4.8 to 5.3 mg C3GE g^−1^ sample for TAC, 30.6 to 69.4 mg GAE g^−1^ sample for TPC, 57.3 to 71.7% inhibition for DPPH, and 498.2 to 579.2 μmol TE g^−1^ sample for FRAP.

The mathematical equations characterising the correlations between the responses and independent variables were obtained by applying regression analysis to the experimental results. All responses (TAC, DPPH, FRAP, and TPC) were fitted to second-order polynomial equations. The generated polynomial equations are listed below.

**TAC** = 4.9236 + 0.1155A + 0.1497B + 0.2247C + 0.0543D + 0.0356AB − 0.0513AC − 0.0111AD + 0.0917BC − 0.0617BD + 0.1317CD + 0.0564A^2^ + 0.1128B^2^ + 0.082C^2^ + 0.0795D^2^

**TPC** = 61.6532 + 5.098A − 2.4681B + 1.7928C + 11.1279D − 7.6667AB + 6.0196AC + 5.758AD + 2.2626BC + 2.956BD + 4.3127CD − 0.2313A^2^ − 4.0819B^2^ − 8.0284C^2^ − 20.5013D^2^

**DPPH** = 60.4344 + 1.8914A + 5.0396B − 1.3923C + 2.8527D + 4.7507AB − 4.9614AC + 1.1289AD + 5.0027BC − 3.0708BD + 1.0084CD + 3.7043A^2^ + 5.7825B^2^ + 7.7996C^2^ + 2.9766D^2^

**FRAP** = 542.1005 + 1.4944A + 24.4853B + 28.3624C + 25.7974D + 0.4453AB + 2.3223AC + 7.6487AD + 2.1843BC + 8.6298BD + 2.6137CD + 5.0947A^2^ − 7.7968B^2^ − 13.6223C^2^ − 11.3648D^2^

The adequacy and goodness of fit of the regression models were then assessed through ANOVA and descriptive statistics (Table 3). The developed models were remarkably significant for all responses, as indicated by low *p*-values (<0.0001) and high F-values (10.945-202.206). The lack-of-fit analysis indicated that variance attributable to pure error was negligible (*p* > 0.05) for all constructed models, confirming that the models adequately fit the data. Additionally, the high coefficients of determination (R^2^: 0.6714–0.9742) indicate good agreement between the model-predicted and experimental data. Moreover, the predicted R^2^ and adjusted R^2^ values (difference < 0.2) were in reasonable agreement, revealing a strong correlation between the experimental and predicted values.

The significance of each independent variable for the responses was assessed through F- and *p*-values derived from ANOVA outcomes. Generally, a higher F-value and a lower *p*-value indicate a greater impact of the corresponding factor, with variables exhibiting *p*-values below 0.05 (at a 95% confidence level) being considered statistically significant for the examined response. Based on ANOVA (Table 3), all responses (TAC, TPC, DPPH, and FRAP) were significantly affected by the linear terms of sonication time (A), extraction temperature (B), solvent-to-solid ratio (C), and NaDES water content (D), except FRAP, which was not influenced by the extraction duration. All variables showed a positive influence on the responses, except for the solvent-to-solid ratio for DPPH and the extraction temperature for TPC, which demonstrated a negative effect. As far as interactive effects are concerned, CD had a significant impact on TAC, and AB, AC, BC, and BD had a significant impact on DPPH response. For TPC, all interactive terms were significant, whereas no interactive effects were observed for FRAP. Among the quadratic effects, all quadratic terms were statistically significant for DPPH, FRAP, and TPC, except for the A^2^ term, which was not significant for FRAP and TPC. For the TAC response, only the B^2^ term was statistically significant.

#### 3.2.2. Responses Surface Analysis

**Figure 3:**
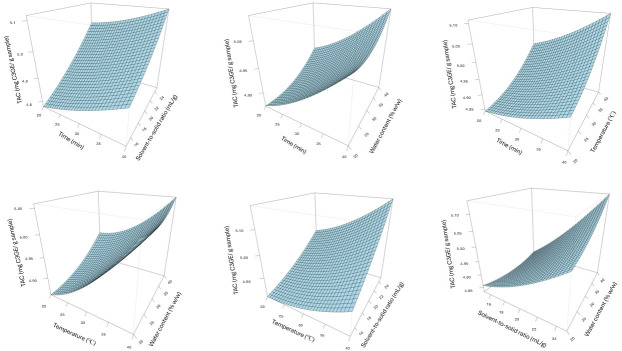
Response surface plots illustrating the interactive impacts of extraction time, extraction temperature, solvent-to-solid ratio, and water content in NaDESs on TAC response.

The predictive polynomial models were used to generate 3D response surface plots to elucidate the interaction between independent variables (Figure 3). These graphs display the impact of two variables on the response simultaneously, while maintaining the remaining parameters at their central coded levels (zero).

The recovery of anthocyanins (TAC), polyphenols (TPC), and antioxidants (DPPH) was positively affected by the processing time. Generally, longer extraction times promote more extensive interactions between the NaDESs and the target compounds, therefore enhancing the mass transfer of analytes into the ChCl-PG media [28,29]. The absence of downward curvature suggests that anthocyanin and phenolic degradations were minimal within the tested duration range.

Extraction temperature demonstrated a differential impact on the measured responses. For TAC, DPPH, and FRAP, a clearly positive effect of processing temperature was observed. Higher temperatures likely enhanced solubility, reduced solvent viscosity, and accelerated mass transfer, facilitating the release of anthocyanins and antioxidants from the plant matrix. In addition, increased temperature may enhance the disruption of plant cells and improve solvent penetration into the matrix [30,31]. However, excessive temperatures can also promote anthocyanin degradation through structural changes in the flavylium cation and oxidation reactions. In contrast, temperature had a negative effect on TPC, with the highest polyphenolic yields observed at lower temperatures. This effect may be attributed to the presence of heat-sensitive phenolics in DR, which can degrade at elevated temperatures [32].

The solvent-to-solid ratio was one of the most influential parameters affecting the responses. Higher ratios improved TAC, FRAP, and TPC values, likely due to the increased solvent availability, facilitating mass transfer and solute diffusivity. The DPPH response showed a nonlinear behaviour, with the lowest values at intermediate solvent-to-solid ratios and higher antioxidant capacity at both low and high ratios. This suggests that low solvent-to-solid ratios improve the interactions between plant antioxidants and NaDES molecules, due to the greater contact surface area, while high ratios maximize the analytes’ diffusion capacity [33,34]. Moreover, the improved extraction efficiency observed at higher solvent-to-solid ratios may be related to the increased availability of solvent molecules for interaction with anthocyanins, leading to improved solubilization and reduced solvent saturation effects [35].

Water content in NaDESs also had a significant impact on all dependent variables. For all examined responses (TAC, TPC, DPPH, and FRAP), higher water levels led to a greater recovery of the targeted analytes. This can be explained by the reduction in the viscosity and the increase in the polarity of the extraction solvent due to the addition of water, resulting in a higher extraction rate [30]. Furthermore, water addition modifies the hydrogen-bonding network and polarity of NaDESs, which may improve anthocyanin solubilization and facilitate analyte diffusion during the extraction process [36].

#### 3.2.3. Multi-Response Optimization and Model Validation

The optimal operating parameters were determined through multi-response optimization using the desirability function method, with criteria established to maximize TAC, TPC, and antioxidant activity (DPPH and FRAP) of DR extracts. The optimal UAE conditions, considering all examined responses equally, were determined as follows: a 40 min extraction time, an extraction temperature of 40 °C, a solvent-to-solid ratio of 25 mL g^−1^, and a water content in NaDES of 40% (*v*/*v*). Under the investigated optimal extraction parameters, the predicted response values were 5.3 mg C3GE g^−1^ sample for TAC, 70.7% inhibition for DPPH, 581.2 μmol TE g^−1^ sample for FRAP, and 64.6 mg GAE g^−1^ sample for TPC, with a desirability index of 0.884. To assess the predictive ability of the developed models, further experiments were carried out under the optimal UAE conditions. The obtained experimental results were compared with the model-predicted values and were in good agreement, with percentage errors ranging from 0.63 to 1.89% (Table 4). The strong degree of correlation between experimental and predicted outcomes demonstrates the high predictive ability of the models, as well as their effectiveness in determining the optimal extraction parameters.

### 3.3. Optimization of the Extraction Procedure of DR Using ANN

#### 3.3.1. ANN Model Construction

ANNs have recently emerged as powerful tools for simulating and optimizing extraction processes, thanks to their strong predictive capabilities, particularly for modeling complex nonlinear relationships. The ANN model was developed using the experimental CCD matrix and consisted of an input layer with four neurons corresponding to the independent variables (extraction time, temperature, solvent-to-solid ratio, and NaDES water content), a hidden layer, and an output layer with four neurons representing the measured responses (TAC, TPC, DPPH inhibition, and FRAP). Because using two or more hidden layers may increase the risk of overfitting, a single hidden-layer network was employed during training. Determining the number of neurons in the hidden layer is also crucial for building ANN models. An ANN model with a small number of hidden neurons can lead to a large training error, while many hidden neurons may cause overfitting, leading to poor generalization performance. Therefore, in the present study, the number of hidden neurons was determined by training different feed-forward networks of various topologies and selecting the one with the lowest MSE and maximum R^2^ values.

For ANN model construction, the experimental data, consisting of 90 points (30 experimental combinations performed in triplicate), were divided into two main subsets: the training/validation set (80%) and the test set (20%). Using k-fold cross-validation, the training/validation set was randomly partitioned into five subsets (k = 5). In each iteration, one subset was used for model validation, while the remaining subsets were employed for training the ANN model. This procedure was repeated ten times with different random partitions to minimize bias introduced by data splitting and to ensure reliable and consistent model evaluation. The test set was used to assess the model’s performance in predicting “unseen” data. Both the input variables and output responses were normalized to the range 0–1 before model construction.

**Figure 4:**
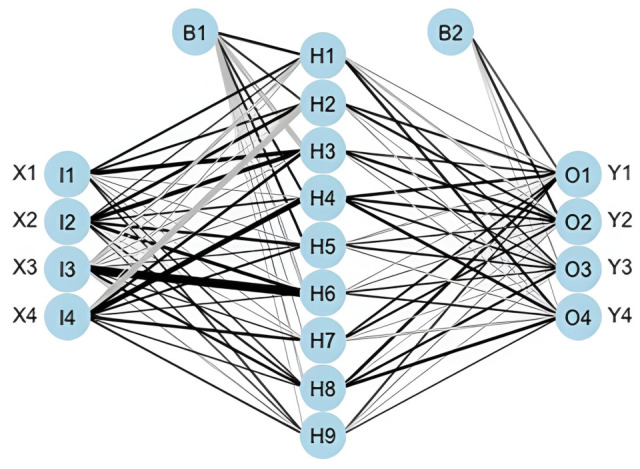
The optimal 4:9:4 architecture of the feed-forward backpropagation ANN model.

The topology of the optimum ANN model (4:9:4), determined based on the minimization of MSE and the maximization of R^2^, using the backpropagation algorithm, is presented in Figure 4. A single hidden layer comprising nine neurons was selected as the optimal network architecture, yielding satisfactory statistical performance. In particular, the MSE values for training, validation, and testing sets were 0.0071, 0.0093, and 0.0134, respectively. The R^2^ between the experimentally obtained and ANN-predicted responses was 0.8793 for the training set, 0.8509 for the validation set, and 0.8175 for the test set. These performance metrics demonstrate the reliability and predictive capability of the developed model across a range of input conditions. The weights and biases of the developed ANN model, used to determine the responses for a given set of processing parameters, are presented in Equations 1–4. In these equations, W^(1)^ and b^(1)^ represent the connection weights and biases between the input neurons and the hidden neurons, respectively, while W^(2)^ and b^(2)^ are the weights and biases connecting the hidden and output neurons.

(1) $$W^{\left(1\right)}=\left[\begin{array}{cccc} 1.3769 & 1.1536 & 0.0909 & -3.1450\\ -0.4977 & 0.4407 & 0.9947 & 2.1066\\ -0.5907 & -5.5479 & -4.2584 & 3.0546\\ 3.0451 & -1.6983 & 1.6503 & 0.9069\\ -0.4378 & 0.4768 & -1.5330 & 4.0898\\ 1.1476 & -0.8006 & 1.6284 & 0.0107\\ 0.9120 & -2.7619 & -1.0150 & 2.2832\\ 7.3403 & 0.0423 & -0.5484 & -0.5428\\ -0.3621 & 0.0497 & 1.5114 & -1.3388 \end{array}\right]$$

(2) $$b^{\left(1\right)}=\left[\begin{array}{ccc} \begin{array}{ccc} \begin{array}{ccc} \begin{array}{ccc} 0.2537 & 0.7500 & 1.3657 \end{array} & 0.1421 & -0.1276 \end{array} & 0.5815 & 0.0775 \end{array} & -1.6103 & 0.5283 \end{array}\right]$$

(3) $$W^{\left(2\right)}=\left[\begin{array}{ccccccccc} 0.2958 & -1.2476 & 0.9703 & 0.4472 & 1.4675 & -2.4061 & -0.2518 & 0.8908 & 1.7270\\ 0.4688 & 1.1128 & -2.2398 & 1.0299 & 1.5268 & 1.0879 & 0.1310 & -1.4979 & 0.9101\\ -0.0154 & -1.1800 & -1.2846 & 0.3306 & -0.3622 & 0.1634 & 0.6261 & 0.6889 & 0.3020\\ -0.2607 & 1.2511 & -0.5278 & -0.0048 & 1.2498 & -1.1189 & -0.5821 & 1.8085 & -1.5053 \end{array}\right]$$

(4) $$b^{\left(2\right)}=\left[\begin{array}{cccc} 0.3778 & -2.3350 & 1.9721 & 0.0822 \end{array}\right]$$

#### 3.3.2. Effect of Extraction Variables on Measured Responses

**Figure 5:**
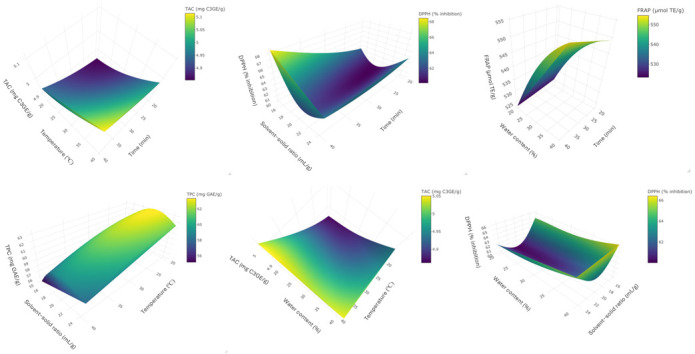
Neural network output surface plots of the combined effects of time, temperature, solvent-to-solid ratio, and water content on total anthocyanin content (TAC), total phenolic content (TPC), 2,2-diphenyl-1-picrylhydrazyl (DPPH) radical inhibition, and ferric reducing antioxidant power (FRAP).

The effects of the input variables on the measured responses, as predicted by the developed ANN model, were graphically demonstrated using three-dimensional response surface plots. Figure 5 illustrates representative effects of the input variables on selected responses, as predicted by the developed ANN model. These plots clearly illustrate the complex and significant effects of the process variables on all measured responses.

Extraction time enhanced the extractability of polyphenols and improved the extracts’ antioxidant capacity. In particular, TAC, TPC, and DPPH responses significantly increased with increasing extraction time due to enhanced solute diffusivity and improved interactions between the target analytes and the solvent [37]. Additionally, prolonged ultrasound irradiation promoted the disruption of plant cell walls, enabling improved solvent penetration into the cells and facilitating the release of polyphenolic antioxidants from the plant matrix [38]. Polyphenol degradation we not observed with prolonged extraction times, in contrast to findings previously reported in the literature [39,40]. In contrast, the FRAP response was not significantly affected by treatment time, reflecting a fundamentally different reaction mechanism from that of the DPPH assay.

The anthocyanin content and the antioxidant activity, expressed as DPPH inhibition and FRAP, were also positively affected by processing temperature. In general, increasing the extraction temperature softens plant tissues and weakens anthocyanin–matrix interactions, thereby enhancing the diffusion rate of anthocyanins and resulting in higher antioxidant activity of the extracts [41]. Furthermore, higher extraction temperatures reduce solvent viscosity, resulting in enhanced solvent penetration into the plant matrix [42]. It is worth noting that the polyphenolic content was negatively affected under high-temperature conditions applied, possibly due to their thermal degradation.

Higher extraction yields and antioxidant activities were achieved at both low and high levels of the solvent-to-solid ratio. Generally, the higher the solvent-to-solid ratio, the higher the extraction yield, due to the greater concentration gradient between the solid matrix and the bulk liquid phase, which serves as the driving force for mass transfer during extraction. However, when the amount of liquid phase relative to the dispersed solid phase is insufficient to ensure adequate mass transfer, equilibrium phenomena may occur, leading to significant resistance to mass transfer. Therefore, selecting an appropriate solvent-to-solid ratio is crucial to ensure sufficient mixing and, consequently, a high diffusion rate of solutes during extraction [43].

Solvent water content also exerted a significant effect on all responses. As observed, increasing the water content of NaDESs improved TAC, DPPH, and FRAP values. In the case of TPC, increasing water content initially enhanced polyphenol extractability, reaching a maximum, followed by a gradual decrease. The addition of water to the extraction solvent reduces the viscosity of the medium, thereby enhancing mass transfer and facilitating solvent diffusion within the plant matrix. At the same time, it also increases solvent polarity, affecting the solubility of the target analytes [37].

#### 3.3.3. Optimization of Extraction Conditions Using a Genetic Algorithm

Once the feed-forward neural network model was developed, a genetic algorithm (GA) was coupled with the ANN model to determine the optimum extraction conditions for achieving the highest yields of total phenolics and anthocyanins, as well as maximum antioxidant activity. GAs can solve nonlinear optimization problems by investigating the entire search space and exploiting promising regions through selection, crossover, and mutation operations applied to individuals within the population. In particular, the GA addresses an optimization process through a four-step cycle, consisting of initializing solution populations (chromosomes), a fitness computation based on the objective function, selecting the best chromosomes, and genetically propagating selected parental chromosomes using genetic operators such as crossover and mutation to create a new population of chromosomes. The process is repeated until an optimal result is achieved [44]. The optimization process was completed after 100 generations of GA.

The hybrid ANN-GA model predicted the following optimal conditions: time of 38.5 min, temperature of 39.4 °C, solvent-to-solid ratio of 24.4 mL g^−1^, and NaDES water content of 39.3% *w*/*w*. Under these optimal extraction conditions, the predicted values for the target responses were as follows: TAC = 5.2 mg C3GE g^−1^, DPPH = 69.9%, FRAP = 574.4 μmol TE g^−1^, and TPC = 63.6 mg GAE g^−1^. Experimental validation under these optimal conditions yielded a TAC of 5.3 ± 0.2 mg C3GE g^−1^ (percentage error = 1.92%), a DPPH of 68.2 ± 0.85% (percentage error = 2.43%), a FRAP of 581.6 ± 8.5 μmol TE g^−1^ (percentage error = 1.25%), and a TPC of 63.4 ± 0.5 mg GAE g^−1^ sample (percentage error = 0.31%), highlighting the strong predictive accuracy of the ANN-GA model.

### 3.4. Comparison Between RSM and ANN Models

The performances of the developed ANN and RSM models in predicting TAC, TPC, DPPH, and FRAP responses were then compared using statistical metrics, including MSE and R^2^ values (Table 1). Similar trends were observed for both models. A direct comparison of MSE and R^2^ values revealed that model performance depends on the response, with no single model consistently outperforming the other. Specifically, the ANN model achieved higher R^2^ and lower MSE values, indicating a closer fit to the experimental data and smaller deviations between predicted and actual responses, particularly for TAC and DPPH. The superior performance of the ANN model in predicting TAC and DPPH responses is likely due to its ability to capture nonlinear relationships among process variables that are not fully captured by the quadratic RSM. In contrast, FRAP and TPC were better described by the RSM model, which achieved slightly higher R^2^ values and lower MSEs for these responses.

The observed differences in model predictive performance can be attributed to the underlying functional relationships between process variables and target responses. Due to the non-parametric nature of ANN models, which allows them to capture complex nonlinear interactions without imposing a predefined equation structure, improved performance was observed for TAC and DPPH responses, where deviations from quadratic behavior are likely more pronounced. In contrast, RSM, which is based on the development of quadratic polynomial models, demonstrated greater predictive ability for FRAP and TPC responses due to their relatively smooth and less complex nature.

The results demonstrate that ANNs, despite their theoretical advantages, do not universally outperform standard regression models, as their performance depends on the degree of system nonlinearity. It is worth mentioning that RSM offers the additional advantage of model interpretability and explicit mathematical relationships among process variables, which are highly beneficial for process understanding and optimization. Overall, both models demonstrated a satisfactory correlation with the experimental data, highlighting their reliability for optimization purposes. These results suggest that while both models are suitable for prediction and optimization, the ANN model provides a practical advantage for responses exhibiting stronger nonlinearity, whereas RSM remains adequate for more linear behaviors.

### 3.5. Phytochemical Analysis and Antioxidant Activity of DR Extract

The optimized extraction of DR bioactive compounds using ChCl-PG NaDES resulted in extracts containing relatively high levels of anthocyanins, polyphenols, flavonoids, proanthocyanidins, and tannins, along with a significant antioxidant capacity. The experimental values obtained under both RSM and ANN optimal conditions are summarized in Table 4.

**Figure 6:**
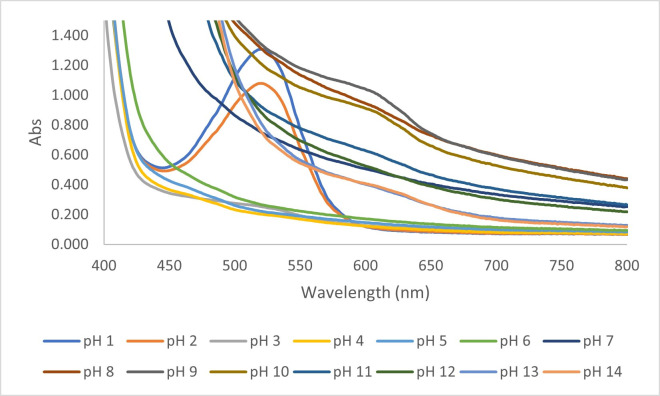
pH sensitivity of optimal DR extract.

The TAC values acquired under optimized extraction parameters were 5.2 ± 0.3 mg C3GE g^−1^ sample (RSM) and 5.3 ± 0.2 mg C3GE g^−1^ sample (ANN). These findings were found to be greater than those reported by Alizadeh and Fattahi (2021) (0.69 to 3.67 mg C3GE g^−1^ sample) and in the same range as those reported by Torusdağ and Bakkalbaşı (2021) (5.16 ± 0.14 mg C3GE g^−1^ sample) [12,45]. These variations in TAC values among studies can be attributed to several factors, including differences in plant material, geographical origin, and extraction conditions applied. In particular, previous studies have shown that anthocyanin content is highly influenced by cultivar, maturity stage, and environmental conditions, as well as by solvent composition and extraction technique, which affect both pigment stability and extraction efficiency [46,47]. Furthermore, the pH-sensitivity of the optimum extracts was investigated, given the ability of anthocyanins to undergo structural transformations as a function of pH. Figure 6 illustrates the UV–Vis spectra of the DR extract at different pH values, with the observed spectral shifts being consistent with the pH-dependent equilibrium of anthocyanins [45,48].

The obtained TPC values suggest the effective extraction capacity of NaDESs. The RSM-optimized extract achieved 64.18 ± 0.79 mg GAE g^−1^ sample, while the ANN-optimized extract yielded 63.35 ± 0.46 mg GAE g^−1^ sample. These values are considerably higher than those typically reported in the literature for DR. Shameh et al. (2018) reported TPC values ranging from 25.13 to 52.01 mg GAE g^−1^ DR petals, while Torusdağ and Bakkalbaşı (2021) reported a TPC value of 43.53 ± 3.25 mg GAE g^−1^ sample, using conventional extraction solvents [12,49]. The enhanced TPC values using NaDESs can be attributed to its unique physicochemical characteristics. The extensive hydrogen-bond interactions between the solvent and phenolics facilitate improved solubilization, promote efficient mass transfer, and enhance extraction efficiency. The superior effectiveness of NaDESs in polyphenolic recovery compared with conventional solvents, attributed to their strong hydrogen-bonding capacity, has already been reported in the literature for various matrices [50,51]. On the other hand, Koraqi et al. (2024), who optimized the UAE of phenolics using the ternary NaDES lactic acid–citric acid–glycerol, reported an even higher TPC value, likely due to different solvent composition [52]. Differences in TPC across studies are influenced by various parameters, including the plant blooming period, geographical origin, and experimental conditions.

The TFC similarly reflected the extraction efficiency of the NaDESs. The RSM and ANN models gave TFC values of 48.79 ± 2.15 mg CE g^−1^ sample and 50.25 ± 2.10 mg CE g^−1^ sample, respectively. Önder et al. (2022) reported a significantly lower TFC value (4.3 mg CE g^−1^ fresh sample) using 80% cold methanol under stirring conditions [53]. Furthermore, several studies express TFC results as quercetin equivalents rather than catechin equivalents, making direct comparisons between the results difficult [12,49]. Nevertheless, the high TFC values obtained in this study highlight the efficiency of NaDES combined with UAE for the extraction of bioactive components from plant materials, which is consistent with previous literature data indicating that NaDESs enhance the solubilization of flavonoids due to their hydrogen-bonding capacity and tunable polarity [54].

The content of proanthocyanidins and hydrolysable tannins were determined in the optimized extracts. The TPAC reached 30.64 ± 2.02 mg CE g^−1^ sample (RSM) and 31.60 ± 0.91 mg CE g^−1^ sample (ANN), while THTC were quantified at 60.68 ± 2.61 mg TAE g^−1^ sample (RSM) and 58.63 ± 1.48 mg TAE g^−1^ sample (ANN), respectively. Literature provides limited data on the individual quantifications of proanthocyanidins and hydrolysable tannins in DR. Under this framework, the results of the present work provide new quantitative insights into these less-characterized compounds. The relatively high values obtained in this study indicate that both tannin subclasses represent a significant proportion of the rose’s phytochemical profile.

Antioxidant activity was evaluated using complementary assays reflecting different mechanisms of action. In particular, the DPPH assay was applied in order to assess the free scavenging capacity of the DR extracts, while the FRAP assay was employed to evaluate their reducing power. The DPPH values were 68.77 ± 3.72% inhibition for the RSM extract and 68.18 ± 0.85% inhibition for the ANN extract. The FRAP values reached 591.86 ± 9.62 μmol TE g^−1^ sample (RSM) and 581.63 ± 8.48 μmol TE g^−1^ sample (ANN). To facilitate comparison, DPPH radical scavenging activity was expressed in Trolox equivalents. The optimized DR extract yielded antioxidant activities of 1651 μmol TE g^−1^ (RSM) and 1636 μmol TE g^−1^ (ANN). Both assays indicate that the extracts exhibit notable antioxidant activity under the studied conditions.

### 3.6. Anthocyanidin Profile of DR Extract and Its Stability

The anthocyanidin composition of the ANN optimum DR extract was determined by HPLC-PDA analysis (Table 5). Among six anthocyanidins analysed, only cyanidin was quantified, with a concentration of 1.07 ± 0.05 mg g^−1^ sample, while pelargonidin was detected at a non-quantifiable level. The remaining four anthocyanidins were not detected in the DR NaDES extract. This finding is consistent with previous studies reporting that cyanidin derivatives constitute the dominant pigments in pink rose petals, whereas other anthocyanins are usually absent or found in trace levels [55,56,57]. The lack of additional anthocyanidins may suggest a high selectivity by the ChCl-PG extractor medium toward the primary pigment fraction. The polarity and hydrogen-bonding network created by ChCl and PG may favour the solubilization and stabilization of cyanidin derivatives compared to other anthocyanins. In addition, the acidic hydrolysis procedure prior to HPLC analysis may have contributed to partial degradation or structural transformation of less stable anthocyanidins. However, it is also possible that other anthocyanidins existed at concentrations below the detection limits of the current analytical method. While degradation during extraction was minimized by the protective supramolecular structure of the NaDESs, the potential for minor constituent loss cannot be entirely ruled out. These results establish cyanidin as the primary bioactive marker for the quality and standardization of the extracts produced in this study.

**Figure 7:**
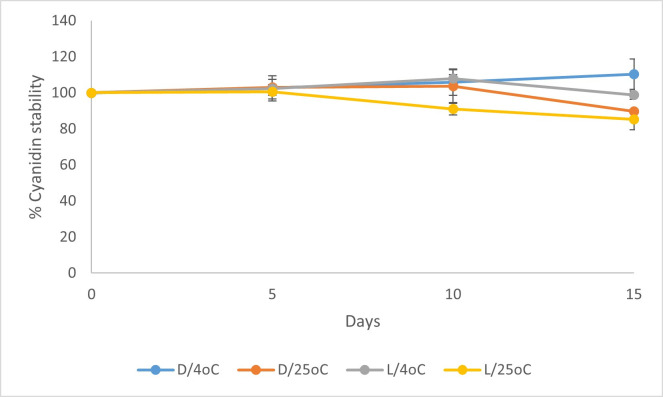
Cyanidin stability in different temperature and light conditions.

The stability of cyanidin in the DR extract was further evaluated under different storage conditions (4 °C vs. 25 °C; dark vs. light). As shown in Figure 7, cyanidin exhibited a higher stability at 4 °C, especially when protected from light exposure. The accelerated degradation observed at 25 °C under light exposure reflects the combined impact of thermal stress and photooxidation, both known to destabilize anthocyanins [58,59]. The enhanced stability observed at lower temperatures in the dark over the 15-day period highlights the protective effect of cold storage and limited light exposure. These findings also suggest that NaDESs may offer partial protection of anthocyanin pigment due to hydrogen-bonding environment among analytes and solvent molecules [35]. In contrast, light exposure and storage at 25 °C resulted in a greater degree of degradation (14.82%). Τhe observed decrease in anthocyanin content over time, particularly at higher temperatures, likely follows well-documented degradation pathways, such as the transformation of the flavylium cation into the colorless chalcone or the subsequent formation of phenolic acids through ring opening. While the NaDES matrix appears to offer a protective effect compared to traditional aqueous systems, the exact nature of the degradation products in these eutectic mixtures remains to be fully elucidated. A comprehensive mapping of these pathways would necessitate the use of advanced Mass Spectrometry (MS) and NMR studies to identify specific structural transitions, which were beyond the initial scope of this investigation. Nonetheless, the current stability data serves as a practical baseline for the shelf-life evaluation of DR extracts.

### 3.7. Antimicrobial Effects of DR Extract and Its Stability

Anthocyanins are recognized as multifunctional agents with potent antioxidant and antimicrobial properties suitable for food preservation [60]. Given that DR extracts have demonstrated significant inhibitory activity against various foodborne pathogens, this study also evaluated its antimicrobial potency [4,61]. Specifically, the efficacy of DR extract was tested against Gram-positive and Gram-negative bacteria, as well as yeasts. Furthermore, the storage stability of these antimicrobial properties was investigated to assess long-term viability. The results indicated that the MIC values for most tested microorganisms were 5% (*v*/*v*) of the DR extract. An exception was noted for *C. albcans*, which required a higher concentration of 15% (*v*/*v*) to achieve inhibitory effects. Additionally, the IC_50_ values determined across all microorganisms ranged from 17% to 27% (*v*/*v*) (Table S3). Therefore, the concentration of 25% *v*/*v* of DR extract was used to screen the stability of antimicrobial potency against a panel of microorganisms for a period of 15 days. At Day 0, a 25% (*v*/*v*) concentration of the DR extract exhibited notable antibacterial activity, with growth inhibition ranging from 69.2% to 82.2%. The Gram-positive pathogens, *S. aureus* (72.9%) and *L. monocytogenes* (72.1%), demonstrated comparable susceptibility to the treatment. Conversely, among the Gram-negative bacteria, *S. enterica* proved more resilient than *E. coli*. Bacteria appeared more sensitive to the DR extract than yeasts under the tested conditions; at the same 25% (*v*/*v*) concentration, growth reduction was limited to 34.2% for *C. albicans* and 62.8% for *S. cerevisiae*. Bacteria are generally more sensitive to anthocyanin extracts than yeasts and fungi. Research across various berry extracts enriched in anthocyanins shows that while anthocyanins significantly inhibit or kill bacterial pathogens, they often have little to no effect on yeast species such as *Candida* spp. or *Saccharomyces* spp. at similar concentrations [62]. This difference is attributed to complex chitin-based cell walls and the eukaryotic structure of yeasts, providing a robust physical and metabolic barrier against anthocyanins.

**Figure 8:**
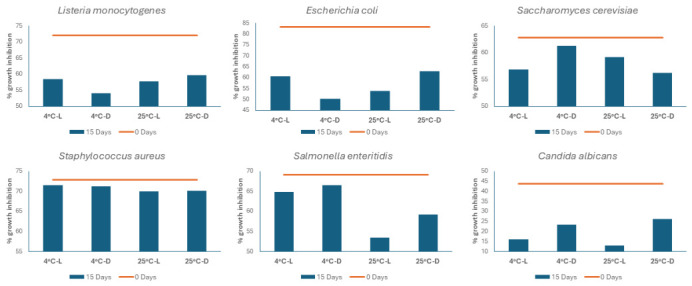
Stability of DR extract antimicrobial potency against foodborne pathogens evaluated at day 0 and day 15 under different light and temperature conditions.Where 4 °C-L: storage at 4 °C under light, 4 °C-D: storage at 4 °C under dark, 25 °C-L: storage at 25 °C under light, 25 °C-D: storage at 25 °C under dark.

In the next phase, the stability of the antimicrobial potential of the DR extract was investigated over a 15-day period under both light and dark conditions at 4 °C and 25 °C. Results revealed that the antimicrobial response varied depending on the microorganism (Figure 8). The inhibitory effect of the DR extract remained relatively stable against *S. aureus* and *S. cerevisiae* throughout the storage period across all tested conditions. In contrast, a 20% reduction in inhibition was observed for *L. monocytogenes* under all treatments, with this decrease becoming evident after 10 days of storage. Regarding Gram-negative bacteria, the inhibitory effect also declined. Light exposure appeared to influence the antimicrobial activity, whereas storage temperature showed no notable effect. A similar trend was observed for *E. coli* and *C. albicans*, though they exhibited a more pronounced loss of activity; specifically, a depletion of 30% and 60% in inhibitory effect was recorded after 15 days of storage under dark and light conditions, respectively. For *C. albicans*, this decline began at the earliest stages of storage. The observed decline in antimicrobial efficacy can be partially attributed to the degradation of anthocyanins within the DR extract during storage; specifically, a reduction of approximately 20% in cyanidin content was recorded (Figure 7). Additionally, the instability of non-anthocyanin constituents, such as flavonoids and terpenes, likely contributed to the decrease in antimicrobial potency. While the synergistic interactions governing the extract’s potency warrant further investigation, such a characterization remains beyond the scope of the present study.

## 4. Conclusions

In the present study, UAE combined with NaDES was applied for the effective recovery of anthocyanins and antioxidant components from DR. Different eco-friendly solvents were assessed, with the ChCl-PG NaDES identified as the most suitable extraction solvent. Extraction parameters were optimized utilizing both RSM and ANN approaches to maximize anthocyanin and phenolic yield and antioxidant activity of the extracts. Both RSM and ANN models produced comparable results and demonstrated a satisfactory correlation between predicted and experimental data. Chromatographic analysis of the optimized extract confirmed cyanidin as the dominant anthocyanidin, a finding consistent with the typical phytochemical profile of this species. Stability evaluations indicated that the recovery of DR extracts is best maintained under cold storage in the absence of light, providing practical guidelines for its handling. Furthermore, the extracts exhibited antimicrobial effects against the tested foodborne bacteria and yeasts. Overall, these findings highlight the potential of NaDES-based green extraction as a sustainable approach for recovering bioactive components from DR flowers. While further studies are needed to evaluate their performance in complex matrices, these results support the possible application of DR extracts as natural pigments, antioxidants, and antimicrobial additives in the food and pharmaceutical industries.

## Figures and Tables

**Table 2:** Central Composite Design of extraction independent variables and experimental responses.

Run	Independent Variables				Dependent Variables			
	Extraction Time (min)	Extraction Temperature (°C)	Solvent-to-Solid Ratio (mL g^−1^)	Water Content (%)	TAC (mg C3GE g^−1^ Sample)	TPC (mg GAE g^−1^ Sample)	DPPH (% Inhibition)	FRAP (μmol TE g^−1^ Sample)
1	20	40	25	40	4.9	61.4	59.8	512.4
2	30	30	20	30	5.0	43.3	61.3	524.5
3	20	20	25	40	4.9	61.7	59.7	539.6
4	30	30	10	30	5.0	57.2	71.7	549.2
5	20	40	15	20	4.9	61.7	59.0	541.8
6	30	30	20	30	5.1	52.2	65.3	559.2
7	30	30	20	30	5.0	52.2	57.3	521.6
8	40	40	15	40	4.9	56.9	65.3	544.0
9	40	20	15	40	5.2	54.1	70.0	561.5
10	10	30	20	30	5.3	63.4	71.0	579.2
11	20	40	15	40	4.9	60.1	60.7	542.4
12	30	50	20	30	4.9	51.4	64.2	498.2
13	20	20	25	20	4.8	50.4	63.5	499.3
14	30	30	30	30	5.3	56.7	65.6	559.1
15	40	40	15	20	5.2	47.5	68.4	546.4
16	40	40	25	40	5.2	57.6	68.9	572.2
17	30	30	20	10	4.8	55.5	60.4	549.1
18	30	30	20	30	5.1	67.9	65.2	550.6
19	40	20	25	40	4.9	60.6	68.3	518.1
20	30	10	20	30	5.2	45.9	68.5	545.8
21	40	20	15	20	4.9	62.7	61.2	544.1
22	50	30	20	30	5.1	69.4	63.4	552.0
23	20	20	15	40	4.8	51.1	68.3	503.1
24	30	30	20	50	5.1	52.3	64.9	543.3
25	30	30	20	30	4.9	30.6	58.9	507.6
26	30	30	20	30	4.8	54.2	66.5	516.1
27	40	20	25	20	5.1	44.5	71.5	514.4
28	40	40	25	20	4.9	46.1	64.0	517.6
29	20	20	15	20	4.9	61.6	61.4	541.3
30	20	40	25	20	5.0	62.4	60.6	543.5

TAC: Total anthocyanin content, TPC: total phenolic content, DPPH: 2,2-diphenyl-1-picrylhydrazyl, FRAP: Ferric reducing antioxidant power, C3GE: Cyanidin-3-glucoside equivalents, GAE: Gallic acid equivalents, TE: Trolox equivalents.

**Table 3:** ANOVA results of TAC, TPC, DPPH, and FRAP models of Damask rose extracts.

Term	TAC		TPC		DPPH		FRAP	
	F-Value	*p*-Value	F-Value	*p*-Value	F-Value	*p*-Value	F-Value	*p*-Value
Model	10.945	<0.0001	202.206	<0.0001	39.815	<0.0001	44.909	<0.0001
A	20.27	<0.0001	232.02	<0.0001	25.46	<0.0001	0.64	0.4274
B	34.09	<0.0001	54.38	<0.0001	180.79	<0.0001	170.95	<0.0001
C	76.74	<0.0001	28.69	<0.0001	13.80	0.0004	229.37	<0.0001
D	4.49	0.0375	1105.49	<0.0001	57.93	<0.0001	189.76	<0.0001
AB	0.32	0.5726	87.46	<0.0001	26.78	<0.0001	0.01	0.9229
AC	0.67	0.4163	53.91	<0.0001	29.20	<0.0001	0.26	0.6142
AD	0.03	0.8605	49.33	<0.0001	1.51	0.2227	2.78	0.0996
BC	2.13	0.1487	7.62	0.0073	29.69	<0.0001	0.23	0.6353
BD	0.97	0.3288	13.00	0.0006	11.19	0.0013	3.54	0.0638
CD	4.39	0.0394	27.67	<0.0001	1.21	0.2756	0.32	0.5705
A^2^	1.38	0.2434	0.14	0.7129	27.91	<0.0001	2.11	0.1501
B^2^	5.53	0.0213	42.50	<0.0001	68.01	<0.0001	4.95	0.0291
C^2^	2.92	0.0915	164.40	<0.0001	123.73	<0.0001	15.12	0.0002
D^2^	2.75	0.1016	1072.06	<0.0001	18.02	<0.0001	10.52	0.0018
Lack of fit	0.0976	0.9998	0.0556	0.9999	0.1540	0.9985	0.0257	1.0000
R^2^	0.6714		0.9742		0.8814		0.8934	
R^2^_adj_	0.6101		0.9694		0.8593		0.8735	
R^2^_pred_	0.5227		0.9613		0.8257		0.8457	

TAC; Total Anthocyanin Content, TPC: Total Phenolic Content, DPPH: 2,2-diphenyl-1-picrylhydrazyl radical scavenging activity, FRAP: Ferric Reducing Antioxidant Power.

**Table 4:** Phenolic content and antioxidant activity of Damask rose extracts obtained under optimal extraction parameters using RSM and ANN optimization approaches.

Analysed Parameter	Model	Experimental Value	Predicted Value	Percentage Error (%)
TAC (mg C3GE g^−1^ sample)	RSM	5.2 ± 0.3	5.3	1.89
	ANN	5.3 ± 0.2	5.2	1.92
DPPH (% Inhibition)	RSM	68.8 ± 3.7	70.7	2.69
	ANN	68.2 ± 0.9	69.9	2.43
FRAP (μmol TE g^−1^ sample)	RSM	591.9 ± 9.6	581.2	1.84
	ANN	581.6 ± 8.5	574.4	1.25
TPC (mg GAE g^−1^ sample)	RSM	64.2 ± 0.8	64.6	0.62
	ANN	63.4 ± 0.5	63.6	0.31
TFC (mg CE g^−1^ sample)	RSM	48.8 ± 2.2	-	-
	ANN	50.3 ± 2.1		
TPAC (mg CE g^−1^ sample)	RSM	30.6 ± 2.0	-	-
	ANN	31.6 ± 0.9		
THTC (mg TAE g^−1^ sample)	RSM	60.9 ± 2.6	-	-
	ANN	58.6 ± 1.5		

TFC: Total flavonoid content, TPAC: Total proanthocyanidin content, THTC: Total Hydrolysable tannin content, CE: catechin equivalents.

**Table 5:** Performance metrics of the developed RSM and ANN models (for normalized data, 0–1).

Response	RSM		ANN	
	R^2^	MSE	R^2^	MSE
TAC	0.6714	0.0176	0.6869	0.0183
TPC	0.9741	0.0010	0.9624	0.0022
DPPH	0.8814	0.0075	0.9019	0.0066
FRAP	0.8934	0.0054	0.8870	0.0064

## Data Availability

The original contributions presented in this study are included in the article/Supplementary Material. Further inquiries can be directed to the corresponding authors.

## Supplementary Materials

The following supporting information can be downloaded from https://www.mdpi.com/article/10.3390/antiox15060656/s1: Figure S1. The FTIR spectra of ChCl-PG NaDES and its initial components; Figure S2. Response surface plots illustrating the interactive impacts of extraction time, extraction temperature, solvent-to-solid ratio, and water content in NaDESs on DPPH response; Figure S3. Response surface plots illustrating the interactive impacts of extraction time, extraction temperature, solvent-to-solid ratio, and water content in NaDESs on FRAP response; Figure S4. Response surface plots illustrating the interactive impacts of extraction time, extraction temperature, solvent-to-solid ratio, and water content in NaDESs on TPC response; Table S1. LODs and LOQs, regression equations, and coefficients of determination; Table S2. Anthocyanidin composition of the Damask rose extract obtained using the Artificial Neural Network (ANN) approach; Table S3. Minimum inhibitory concentration (MIC) and 50% growth inhibitory concentration (IC50) of Rosa damascena extract on selected microorganisms.

## Abbreviations

The following abbreviations are used in this manuscript:

ACN	Acetonitrle
Ala	Alanine
ANN	Artificial Neural Network
Bet	Betaine
CA	Citric Acid
CE	Catechin Equivalents
ChCl	Choline Chloride
C3GE	Cyanidin-3-glucoside Equivalents
DPPH	2,2-diphenyl-1-picrylhydrazyl
DR	Damask Rose
EtOH	Ethanol
Fru	D-(−)-Fructose
GAE	Gallic Acid Equivalents
Gly	Glycerol
Glyc	Glycine
Glu	D-(+)-Glucose
HBA	Hydrogen Bond Acceptor
HBD	Hydrogen Bond Donor
LA	L-(+)-Lactic Acid
MA	Malic Acid
MeOH	Methanol
NADES	Natural Deep Eutectic Solvent
OA	Oxalic Acid
PG	Propylene Glycol
Pro	Proline
RSM	Response Surface Methodology
Sor	Sorbitol
Suc	Sucrose
TA	Tartaric Acid
TAC	Total Anthocyanin Content
TE	Trolox Equivalents
TFA	Trifluoroacetic Acid
TFC	Total Flavonoid Content
THTC	Total Hydrolyzable Tannin Content
TPC	Total Phenolic Content
TPTZ	2,4,6-tris(2-pyridyl)-s-triazine
UAE	Ultrasound Assisted Extraction
Xyl	Xylitol
