# Supplementary material for: RSM- and ANN-Based Optimization of Bioactive Anthocyanin and Phenolic Compound Recovery from Rosa damascena Flowers Using Natural Deep Eutectic
Source: Antioxidants (Basel). 2026 May 22;15(6):656. doi: 10.3390/antiox15060656 (PMC13296296; doi:10.3390/antiox15060656)
Supplement: Supplementary file 1 [file antioxidants-15-00656-s001.zip › antioxidants-4268384-supplementary.pdf]

Table S1. LODs and LOQs, regression equations and coefficients of determination.

Compounds	t_R (min)	Linear range (mg mL$^{-1)}$	Regression equation	Correlation coefficient (R^2)	LOD (µg mL^{-1})	LOQ (µg mL^{-1})
Delphinidin	13.908 ± 0.046	0.0001-0.01	$y=4*10^7x-2082.3$	0.9997	0.147	0.447
Cyanidin	18.419 ± 0.018	0.0001-0.01	$y=2*10^7x-678.56$	0.9995	0.163	0.494
Petunidin	20.143 ± 0.025	0.0001-0.01	$y=9*10^7x-5652.9$	0.9999	0.102	0.309
Pelargonidin	23.258 ± 0.028	0.0001-0.01	$y=4*10^7x-2401.8$	0.9999	0.116	0.353
Peonidin	25.190 ± 0.005	0.0001-0.01	$y=1*10^8x-2739.7$	0.9999	0.082	0.249
Malvidin	26.383 ± 0.006	0.0001-0.01	$y=9*10^7x-5345.9$	0.9999	0.103	0.311

LOD: Limit of Detection; LOQ: Limit of Quantitation

Table S2. Anthocyanidin composition of Damask rose extract obtained using Artificial Neural Network (ANN) approach.

**Anthocyanidins**	**Concentration (mg g^{-1} sample)**
Delphinidin	ND
Cyanidin	1.07 ± 0.05
Petunidin	ND
Pelargonidin	NQ
Peonidin	ND
Malvidin	ND

Table S3. Minimum inhibitory concentration (MIC) and 50% growth inhibitory concentration (IC_{50}) of *Rosa damascena* extract on selected microorganisms.

	Escherichia coli	*Listeria monocytogenes*	*Salmonella enterica*	*Staphylococcus aureus*	*Candida albicans*	*Saccharomyces cerevisiae*
MIC (% v/v)	5	5	5	5	15	5
IC_{50} (%v/v)	15	22	20	21	27	21

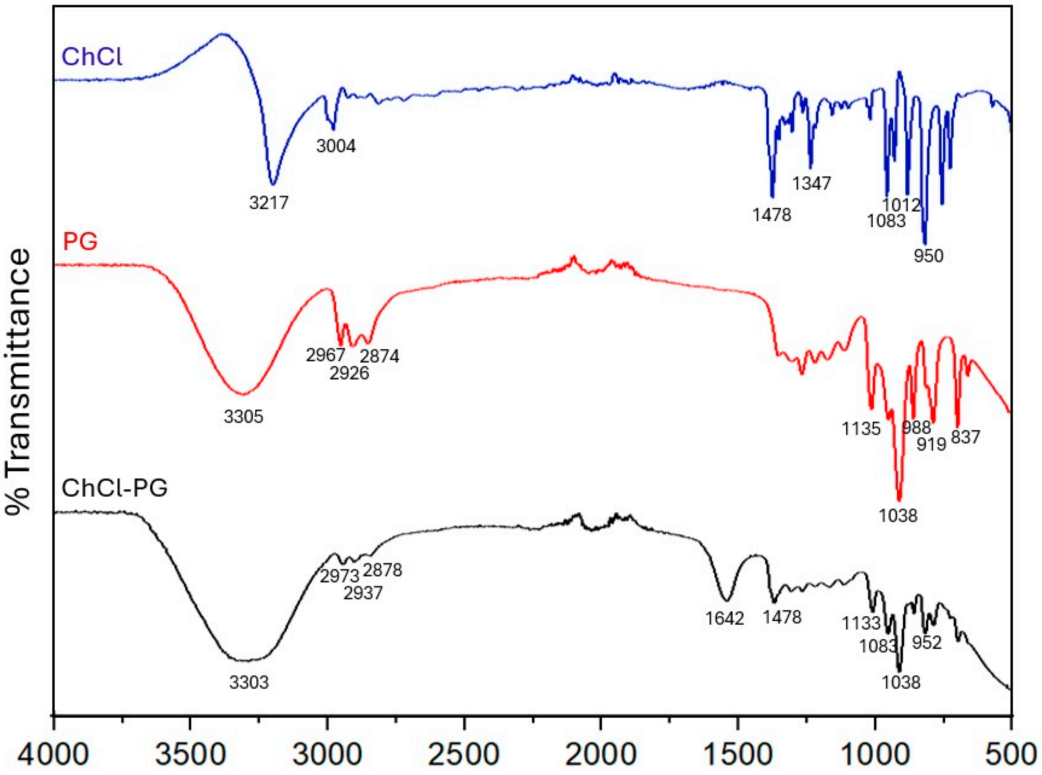

Figure S1. The FTIR spectra of ChCl-PG NaDES and its initial components.

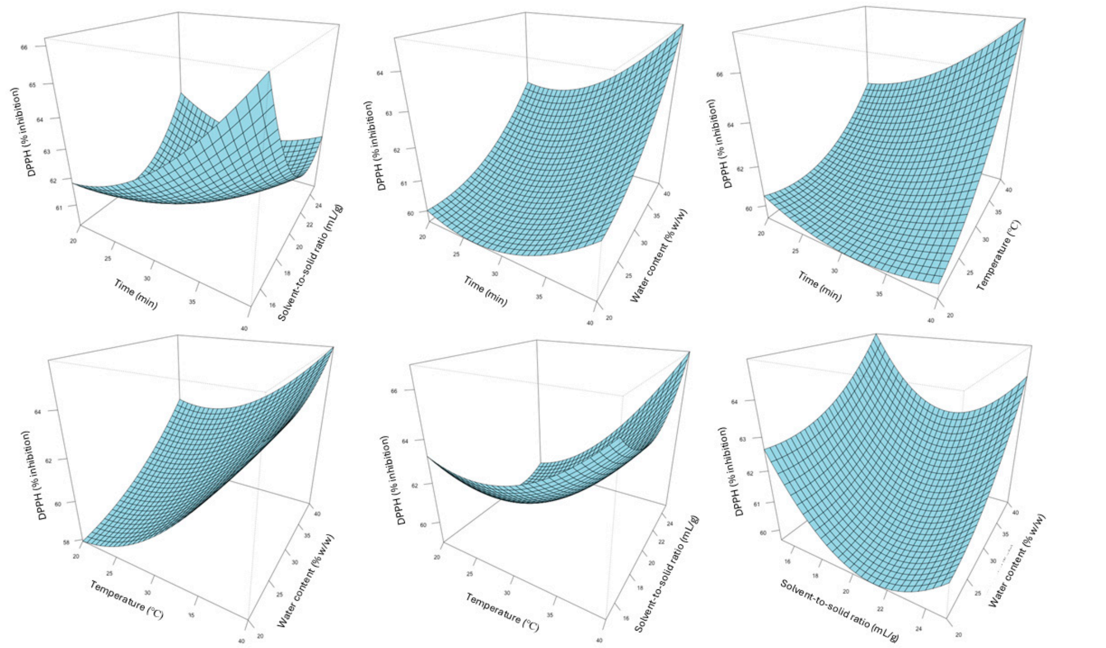

Figure S2. Response surface plots illustrating the interactive impacts of extraction time, extraction temperature, solvent-to-solid ratio, and water content in NaDES on DPPH response.

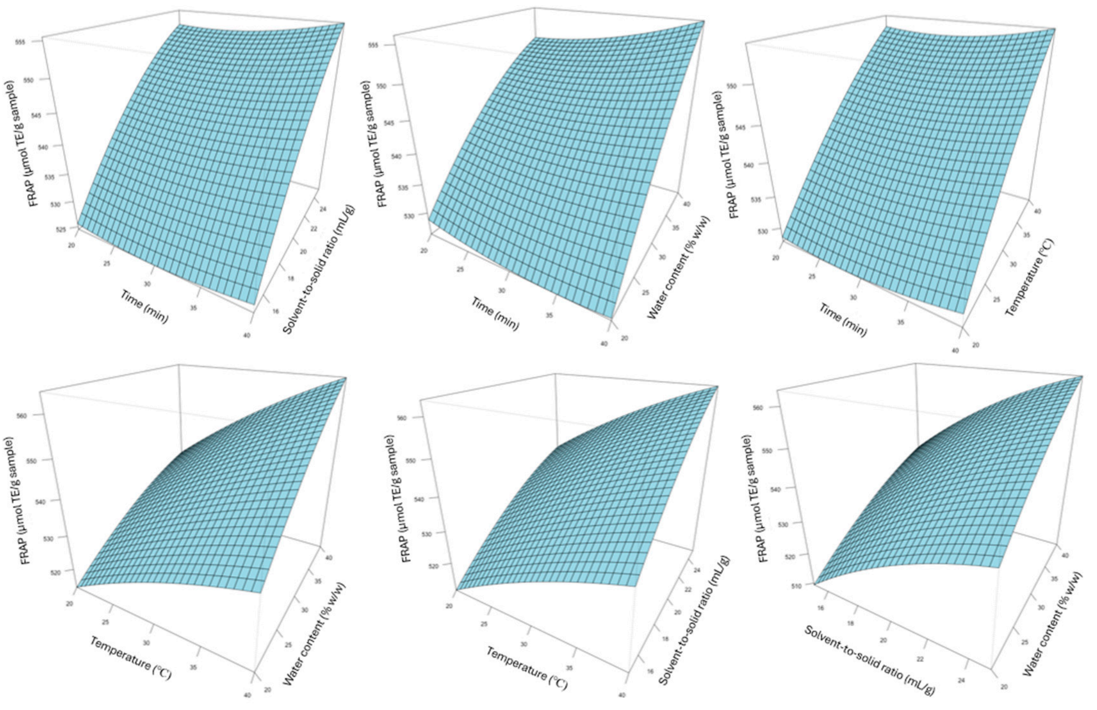

Figure S3. Response surface plots illustrating the interactive impacts of extraction time, extraction temperature, solvent-to-solid ratio, and water content in NaDES on FRAP response.

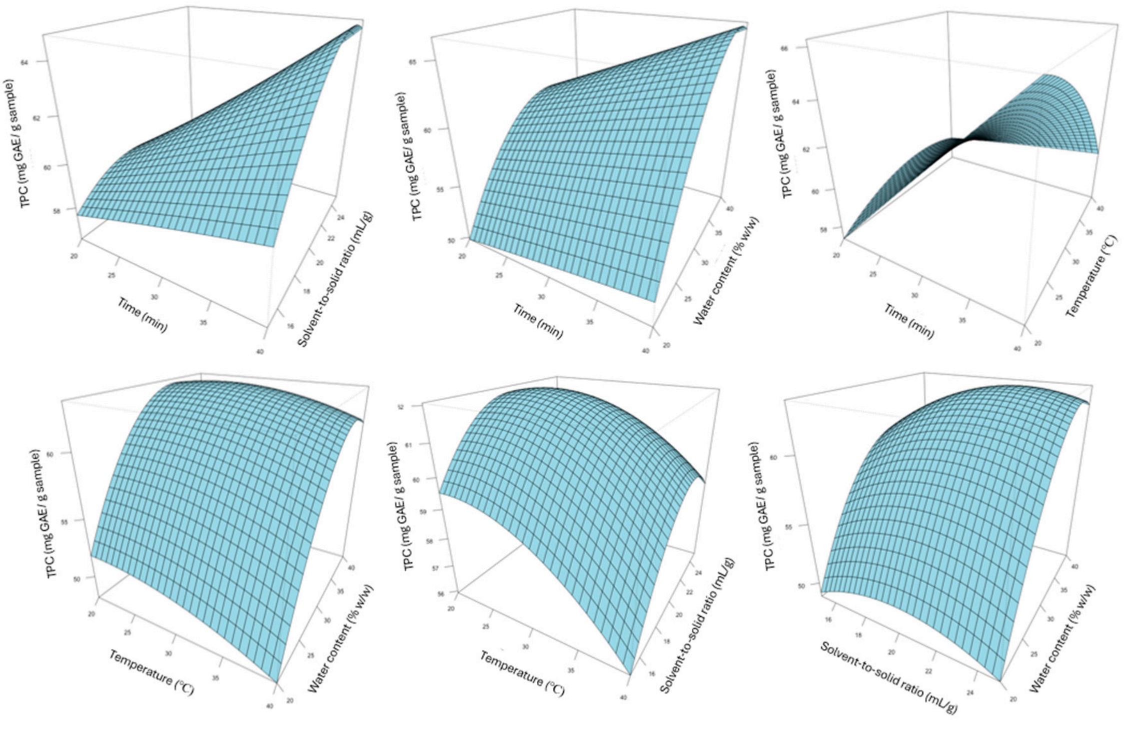

Figure S4. Response surface plots illustrating the interactive impacts of extraction time, extraction temperature, solvent-to-solid ratio, and water content in NaDES on TPC response.
